# The Community Readiness Instrument: A Quantitative Measurement Using Statistical Best Practices to Assess Systemic Change Readiness

**DOI:** 10.3390/bs16010153

**Published:** 2026-01-22

**Authors:** Natalie M. Ricciutti, Jenny L. Cureton, Sijia Zhang

**Affiliations:** 1 Department of Counseling, University of North Carolina Charlotte, 9201 University City Blvd, Charlotte, NC 28223, USA; 2 Counselor Education and Supervision, Kent State University, Kent, OH 44240, USA; jcureton@kent.edu; 3 Department of Educational Leadership, University of North Carolina Charlotte, Charlotte, NC 28223, USA; szhang26@charlotte.edu

**Keywords:** community readiness, substance use, full-scale instrument testing, Rasch analysis, prevention

## Abstract

**Background:** Community readiness assessment is used to gauge a community’s ability to address systemic issues and inform action. The Community Readiness Instrument (CRI) is the only published tool to have undergone rigorous development and testing. The purpose of this study is to further refine the CRI and establish its score reliability and validity evidence so that healthcare professionals, community advocates, and researchers have a strong assessment of community readiness. **Methods:** The present study details continued assessment of the CRI through full-scale testing. We conducted a second-order confirmatory factor analysis to analyze the CRI’s six-factor structure. We also conducted Rasch analyses to determine the item-level fit statistics for each subscale. **Results:** Our results suggest that the CRI is a well-structured quantitative tool with items demonstrating sufficient fit under each first-order latent factor. The items each fell into one-factor solutions, and the six subscales collectively formed a higher-order construct of *Community Readiness.* The CRI continues to demonstrate strong psychometric properties, score reliability, and validity evidence. **Conclusions:** Mental health and addiction professionals can use the CRI to explore change readiness toward a specific issue in their communities. Implications for practitioners, community advocates, and future researchers are provided.

## 1. Introduction

Readiness to change is a well-established concept in the helping professions. Introduced by Prochaska and DiClemente (1982) in the Transtheoretical Model of Change (TTM), it supports researchers and clinicians as they consider an individual’s motivation to change their behaviors. The concept states that an individual is unlikely to make positive behavioral change until they are ready to take action on a problem. Professionals have used change readiness for various treatment issues (Hunter, 2024; Miller & Rollnick, 2023), including substance use and addictions (Chang et al., 2024; Zurawel et al., 2024), abusive relationships (Cunha et al., 2024; McDonagh et al., 2023), and suicide (Sokol et al., 2024; Van Zanden & Bliokas, 2022). Professionals often assess a client’s change readiness at the beginning of treatment and choose interventions that match the individual’s readiness level.

Comparatively, community readiness refers to readiness to address a systemic issue impacting a community (R. W. Edwards et al., 2000; Oetting et al., 1995; Plested et al., 2016; Schröder et al., 2022; Wainwright et al., 2024), such as widespread gun violence, substance use, or high suicide rates. Community change is unlikely to occur until the community as a whole is ready to take action. Therefore, assessing community change readiness is considered a vital step for instituting community-wide efforts (National Institute of Health [NIH], 2011; Substance Abuse and Mental Health Services Administration [SAMHSA], 2022a; World Health Organization [WHO], 2021).

The Community Readiness Instrument (CRI; S. Zhang et al., 2025) is a new assessment tool that measures community change readiness. Initial pilot tests indicated that the CRI has promising psychometric properties and is productive at measuring the construct of *Community Readiness*. The current article begins with a review of existing community readiness assessment tools, including the CRI, specifically comparing evidence of reliability and validity, comprehensiveness, and feasibility of use. We then present methods and results of a full-scale study of the CRI and its factor structure. This includes a second-order Confirmatory Factor Analysis, Rasch Analysis, and Differential Item Functioning analysis for age and primary role subgroups. Finally, we provide recommendations for future use to inform mental health and substance use treatment, prevention, and research.

### 1.1. Community Change Readiness

The concept of readiness to change was introduced within the Transtheoretical Model of Change (Prochaska & DiClemente, 1982) and first applied to individual substance use behaviors (Prochaska & DiClemente, 1983). The model includes the Stages of Change an individual must progress through to create long-term, positive change. The stages include *Precontemplation* (no awareness of the issue and/or no intent to change), *Contemplation* (increased awareness of the issue and considering making changes), *Preparation* (preparing to make changes), *Action* (overt modification of behaviors), *Maintenance* (established changes sustained for at least six months), and *Relapse* (return to previous behaviors). Researchers have utilized these conceptual categories to create and refine several measurements, such as the Readiness to Change Questionnaire, the Stages of Change Readiness and Treatment Eagerness Scale (Miller & Tonigan, 1996), and the University of Rhode Island Change Assessment (McConnaughy et al., 1983). This process has involved instrument development procedures, resulting in longstanding measures that have become standard practice to conceptualize a patient’s likelihood of making positive changes toward a variety of problems (Cunha et al., 2024; Miller & Rollnick, 2023; Sokol et al., 2024).

In comparison, community readiness focuses on a group’s collective readiness to address systemic issues (R. W. Edwards et al., 2000; Oetting et al., 1995; Plested et al., 2016; Standerfer et al., 2022), acknowledging sociocultural factors and norms that impact its likelihood to change (Arambula Solomon et al., 2023; Chilenski et al., 2007; Niknam et al., 2023; Weiner et al., 2008). For example, a community may struggle to address widespread alcohol use if few other activities are available and drinking is an expected norm for many of its subgroups. Community leaders and advocates need a tool to assess systemic readiness toward the issue to create change efforts targeted to the community’s circumstances.

### 1.2. Community Readiness Assessments

Determining a community’s readiness to change an issue is a necessary step in the systemic change process (Kehl et al., 2021; Standerfer et al., 2022). The Community Readiness Model (CRM; Oetting et al., 1995; Plested et al., 2016; Tri-Ethnic Center for Prevention Research [TEC], 2014, 2025) was the first published framework to guide appraisal of community readiness. Informed by the TTM (Prochaska & DiClemente, 1982), its users conduct a community readiness assessment (CRA) involving interviews with representatives from diverse community sectors (Oetting et al., 1995; Plested et al., 2016; Wainwright et al., 2024). By adopting a preventionist approach, community leaders and advocates engage in action planning meetings to determine steps for increasing community change readiness. They then align community efforts toward the target issue with the community’s stage of change (Chilenski et al., 2007; Plested et al., 2016; Schröder et al., 2022; Tri-Ethnic Center for Prevention Research [TEC], 2014, 2025; Wainwright et al., 2024).

The CRM’s major strengths are its focus on culturally informed change and its adaptability to a variety of issues and community types (Kellner et al., 2023; Kostadinov et al., 2015; Schröder et al., 2022). For these reasons, the CRM has been used in over 50 published studies (Kellner et al., 2023), with the most common target issues being substance use and addiction (e.g., Chang et al., 2024; Ghahremani et al., 2021; Cureton et al., 2022), suicide prevention (e.g., Cureton et al., 2023; Allen et al., 2009), and obesity (e.g., Heath et al., 2020; Schröder et al., 2022). International and national organizations have also encouraged using the CRM to support the development of healthy communities. SAMHSA included it in their Evidence-Based Practices Resources Center (https://www.samhsa.gov/libraries/evidence-based-practices-resource-center, accessed on 23 November 2022) and Professional Competencies Guide (Substance Abuse and Mental Health Services Administration [SAMHSA], 2022b). WHO recommended assessing community readiness to increase effectiveness of COVID-19 vaccination programs (World Health Organization [WHO], 2021) and to decrease the prevalence of adverse childhood experiences (World Health Organization [WHO], 2020). NIH included the CRM in their Principles of Community Engagement (2011) as a recommended tool for researchers.

Despite the CRM’s many strengths, it has a number of limitations. First, the CRM does not contain a statistical instrument (Tri-Ethnic Center for Prevention Research [TEC], 2014); thus, its associated CRA lacks the evidence of reliability and validity expected of quantitative measurements. Instead, CRA scoring involves counting and averaging, and its construct validity is determined through hypothesis testing and inter-rater reliability across co-scorers. Second, the data collected to assess the community’s readiness comes from six to eight participants via individual interviews lasting 30–60 min (Kellner et al., 2023; Plested et al., 2016; Tri-Ethnic Center for Prevention Research [TEC], 2014). This small sample size is not sufficient to be representative of most communities (Muellmann et al., 2021). Such limitations have prompted calls for a tool that maintains the strengths of the CRM while being psychometrically sound (e.g., shows evidence of score reliability and validity; Cureton et al., 2022; Hicks, 2019). Such an instrument would allow professionals to (a) quantify and compare change readiness for both pre- and post-prevention efforts and between communities (Kelley et al., 2018; S. Zhang et al., 2025); (b) generalize results to similar communities, populations, and issues (Kesten et al., 2015; Schröder et al., 2022; Wells et al., 2020); and (c) provide statistical data to support applications for future funding (Cureton et al., 2022; Kostadinov et al., 2015).

Table 1 displays tools developed in response to these calls and highlights their attributes of psychometric validation, theoretical grounding, and logistical and conceptual usability. Rigorous testing of quantitative measures typically involves exploratory and confirmatory factor analyses (EFA and CFA) and Rasch analysis to establish score reliability and validity evidence using one or more samples (Bond & Fox, 2015; Frost et al., 2007; Tabachnick & Fidell, 2019; White et al., 2022). Theoretical grounding represents the use of foundational readiness frameworks to inform tool design (i.e., TTM and CRM). SUD represents previous application to substance use disorder or a similar issue, given the readiness concept’s origin for such applications. Finally, issue adaptability refers to the tool’s adaptability to issues other than those for which it was originally developed.

The Campus Community Readiness Survey (CCRS; Hicks, 2019) is based on the CRM and theoretically grounded in the TTM. However, it lacks the statistical rigor needed for generalizability, such as demonstrated score reliability, validity evidence, and measure stability through testing large, representative samples (Prieto & Delgado, 2010). The Readiness Assessment for the Prevention of Child Maltreatment (RAP-CM; Mikton et al., 2013 and the RAP-CM Short Version (WHO, 2013) were developed to assess a community’s or country’s readiness to implement child maltreatment prevention programs. These tools strongly correlate with each other (0.70–0.90; WHO, 2013); however, they are limited to child maltreatment applications (Eissa et al., 2019; Nhassengo et al., 2025, and neither has been extensively used in published research literature. This may be due to the lack of rigorous testing to determine independent psychometric properties of the RAP-CM and RAP-CM Short Version. Psychometric testing of the Systems of Care Readiness and Implementation Measurement Scale (SOC-RIMS; Rosas et al., 2016) showed strong score reliability and evidence of validity to measure readiness in communities with systems of care policies. However, it is not theoretically grounded in the TTM, conceptually aligned with the CRM, nor has it recently appeared in published research.

The Community Readiness Survey (CRS; Beebe et al., 2001) is the only tool grounded in the TTM with published tests demonstrating promising psychometric properties. However, the CRS is an issue-specific tool for adolescent substance use: all items target this issue with phrasing that is not easily adaptable to other issues. For this reason, the CRS is not suitable for assessing community readiness to address any concerns other than adolescent substance use. At this time, community readiness measures such as the CRS (Beebe et al., 2001), CCRS (Hicks, 2019), RAP-CM (Mikton et al., 2013, and the SOC-RIMS (Rosas et al., 2016) appear to fall short in their continued relevance, statistical rigor, theoretical grounding, and adaptability to a variety of community issues. Concerned communities and researchers still need a solid quantitative instrument to assess systemic change readiness within and across communities and time periods. An instrument compatible with the CRM and TTM is needed to achieve rich, comprehensive mixed-methods data and better inform prevention efforts.

### 1.3. Community Readiness Instrument

S. Zhang et al. (2025) created the CRI to address the lack of a statistically and conceptually sound measure of community change readiness. The CRM (Plested et al., 2016; Tri-Ethnic Center for Prevention Research [TEC], 2014) and the TTM (Prochaska & DiClemente, 1982) informed the CRI’s development. Consistent with both frameworks’ origin in substance use prevention, the CRI’s pilot testing targeted the issue of college student substance use within a university community. However, the overall instrument and individual items were designed for broad application to a variety of communities and issues (i.e., simple word replacements) to retain the feasibility of the CRM and TTM.

S. Zhang et al.’s (2025) pilot study included the CRI’s initial development, expert review, testing process, and promising psychometric properties. Further testing using confirmatory and advanced analyses is necessary to determine whether the CRI’s factor structure, latent construct, and fit statistics show adequate statistical strength for measuring community readiness. Thus, our purpose for the current study was to further determine the CRI’s psychometric properties and advance the validity evidence using second-order confirmatory factor analysis (CFA) and Rasch analysis. Our overarching aim was to evaluate and refine the CRI (S. Zhang et al., 2025) so that mental health and addiction professionals, community advocates, and researchers can have an easy-to-use, psychometrically strong tool to sufficiently measure community readiness to change issues that impact community members. We chose to address the following research questions:

1. What is the reliability of the CRI subscale scores with a sample of university students?
2. What are the psychometric properties of the CRI using a second-order confirmatory factor analysis?
3. What are the psychometric properties of the CRI using Rasch analysis?

## 2. Methodology

### 2.1. Participants and Recruitment Procedures

In alignment with CRM recommendations (Plested et al., 2016; Tri-Ethnic Center for Prevention Research [TEC], 2014) and previous community readiness research (Cureton et al., 2022; Kellner et al., 2023; S. Zhang et al., 2025), we defined the issue and the community along with ideal representation across community roles. The target issue was college substance use, and the target community was a public university in the southeastern United States (U.S.). Our sample included students, faculty, and staff, all of whom were at least 18 years old and active members of the university community. Active membership was defined as being enrolled at or employed by the university at the time of participation. Those who were not actively associated with the university (i.e., alumni, individuals living near the university, retired faculty, etc.) were not invited to participate.

Data collection began in January 2024 and concluded in March 2024. Participants were recruited via the university’s email listserv. The emails included a study description, participation instructions, a link to the study in Qualtrics, and the opportunity to enter a draw for one of ten $50 Amazon gift cards. After clicking the link, participants were invited to read and agree to the informed consent. Next, participants viewed instructions for the CRI, including definitions of the target community and issue. Participants were administered the CRI subscales in the following order: *Efforts, Knowledge of Efforts, Leadership, Community Climate, Knowledge of Issue,* and *Resources.* This was followed by a demographic survey, and then a list of substance use and mental health resources in the university and surrounding area was provided. Finally, participants who completed the full study could provide an email address for the gift card drawing.

A total of 891 individuals started the study. Those with at least 20% of data missing were excluded. Initial analyses to detect disingenuous responses or outliers (Dimitrov, 2012) revealed no additional participants to remove. The resultant sample was 496 participants. Regarding age, 256 (51.61%) participants were between 18 and 24 years old, 108 (21.77%) were 25 to 44 years old, 33 (6.65%) were 45 to 64 years old, two (0.4%) were 65 to 84 years old, and 97 (19.56%) did not report their age. Regarding gender, 254 (51.21%) participants identified as female, 119 (23.99%) as male, 15 (5.07%) as non-binary/third gender, four (0.81%) as transgender, and one (0.2%) as gender fluid. One participant (0.2%) selected “not listed/other,” eight (1.61%) selected “prefer not to say,” and 94 (18.95%) did not report their gender identity.

Regarding race, 267 (53.83%) participants identified as White/Caucasian, 59 (11.9%) as African American/Black, 55 (11.09%) as Asian American, 10 (2.01%) as American Indian/Alaskan Native, and two (0.4%) as Native Hawaiian/Pacific Islander. Of the remaining, 16 (3.23%) selected “not listed/other,” 17 (3.43%) selected “prefer not to say,” and 70 (14.11%) did not report their race. Regarding ethnicity, 320 (64.52%) participants identified as Not Hispanic/Latino/a/x/e, 50 (10.1%) as Hispanic/Latino/a/x/e, 16 (3.23%) indicated that their ethnicities were “not listed/other,” and 110 (22.2%) did not report their ethnicity.

We asked participants about their highest level of completed education. Many of the participants (*n* = 194; 39.11%) reported having earned a high school diploma/GED, whereas 97 (19.56%) indicated they had earned a Bachelor’s Degree, 64 (12.95%) a Master’s Degree, and 10 (2.02%) a Doctoral Degree. Of the remaining, 35 (7.06%) selected “other,” one (0.2%) reported that they did not have a high school diploma/GED, and 95 (19.15%) did not report their highest level of completed education. We also asked participants about their primary role in the university community, defined for them as the role in which they spend the most time. For example, a student with an on-campus job would identify as “student,” and a staff member enrolled in courses part-time would identify as “staff.” The majority of participants (*n* = 253, 51.01%) were undergraduate students, 80 (16.13%) were graduate students, 38 (7.66%) were staff, 22 (4.44%) were faculty, and six (1.21%) were certificate students. Of the remaining, three (0.6) indicated they held another role, and 94 (18.95%) did not report their primary role. Among student participants, 62 (18.29%) were freshmen, 44 (12.98%) were sophomores, 72 (21.24%) were juniors, 75 (22.12%) were seniors, 59 (17.40%) were Master’s students, 21 (6.19%) were doctoral students, and six (1.77%) were pursuing a certificate.

### 2.2. The Community Readiness Instrument

The CRI is an assessment of community readiness to change a specific issue. As described by S. Zhang et al. (2025), initial development and testing were informed by the CRM (Plested et al., 2016; Tri-Ethnic Center for Prevention Research [TEC], 2014), the TTM (Prochaska & DiClemente, 1982), and an expert review process. We overviewed its structure and properties here for application to the current follow-up study.

The CRI contains six subscales, representing the six CRM readiness dimensions or influential facets of community readiness (Tri-Ethnic Center for Prevention Research [TEC], 2025): *Efforts, Knowledge of Efforts, Leadership, Community Climate, Knowledge of Issue,* and *Resources.* Each subscale includes nine to 13 items scored on a range of 1 (*strongly disagree*) to 4 (*strongly agree*), representing Stages of Change (Prochaska & DiClemente, 1982). As with TTM scales (DiClemente et al., 2004) and the CRM (Plested et al., 2016), this involved the application of categorical stages to create a continuous interval scale, initial and full-scale testing, and ongoing refinement.

A score of 1 on the CRI indicates *Precontemplation*, 2 indicates *Contemplation*, 3 indicates *Preparation*, and 4 indicates *Action*. The TTM stages of *Maintenance* (sustained change over time) and *Relapse* (regression to prior behavior) do not appear as scores on the CRI range (S. Zhang et al., 2025). The only difference between *Action* and *Maintenance* is sustained change over time, while the only difference between *Maintenance* and *Relapse* is the return to previous behavior. Thus, *Maintenance* could be determined through repeated results (e.g., every six months) that show a community has remained in *Action*; similarly, follow-up results indicating a drop from *Action* to a lower score could indicate *Relapse*. S. Zhang et al. (2025) determined that the inclusion of CRI items that would attempt to account for *Maintenance* and *Relapse* could increase participant confusion. Further, *Relapse* or similar concepts do not appear in the current or previous versions of the CRM (Plested et al., 2016; Tri-Ethnic Center for Prevention Research [TEC], 2014).

Upon administration, the CRI contained 66 items, including 19 opposite-statement items used as a validity check to minimize participants’ automatic responding (Weijters & Baumgartner, 2012). The CRI’s initial testing occurred with 602 students, faculty, and staff in the same university community as the current study and targeted the same issue: college student substance use. All items averaged above 2.0, with the highest-rated item in the *Knowledge of Issue* subscale (*M* = 3.288, *SD* = 0.600) and the lowest-rated item in the *Knowledge of Efforts* subscale (*M* = 2.144, *SD* = 0.793). Results showed promising psychometric properties (S. Zhang et al., 2025): all 47 CRI items fell into one-factor solutions, indicating they measured the underlying constructs of community readiness. McDonald’s Omega values were considered “great” to “excellent” (Dunn et al., 2014; Duran & Celik, 2025; Hayes & Coutts, 2020; McDonald, 1999): *Efforts* ω = 0.89 (95% CI = 0.86, 0.91), *Knowledge of Efforts* ω = 0.91 (95% CI = 0.89, 0.92), *Leadership* ω = 0.94 (95% CI = 0.93, 0.95), *Community Climate* ω = 0.91 (95% CI = 0.89, 0.93), *Knowledge of Issue* *ω* = 0.85 (95% CI = 0.82, 0.87), and *Resources* *ω* = 0.88 (95% CI = 0.86, 0.90).

S. Zhang et al. (2025) also found that the CRI appears to measure the overall concept of *Community Readiness* and the six dimensions that influence community readiness. The second-order CFA confirmed that all items strongly loaded onto each of the six subscales and that each subscale loaded strongly on the higher-order latent factor of *Community Readiness.* This was based on the adequate fit of a six-factor solution: root mean square error of approximation (*RMSEA*) = 0.080, χ2/df = 3.541, standardized root mean square residual (*SRMR*) = 0.080, comparative fit index (*CFI*) = 0.968, Tucker–Lewis Index (*TLI*) = 0.967, and goodness of fit (*GFI*) = 0.75 (MacCallum et al., 1999. Initial Rasch analysis results for the six CRI first-order latent factors indicated that each item productively measured its subscale construct. All items’ Infit and Outfit statistics fell within the “productive” range of 0.50 and 1.50.

Whereas the initial CRI used community-specific phrasing for each item (e.g., “[XU] community members seem troubled by student substance use”), we altered the items slightly in the present study (e.g., “Community members seem troubled by student substance use”). The aim of the alteration was to promote adaptability to a variety of communities through community-neutral language at the item level. In both versions, community-specific phrasing appeared in brief introductions to each subscale. This change is unlikely to impact the CRI’s psychometric properties, as it does not change the construct being measured (Coons et al., 2009).

### 2.3. Demographics

The demographic questionnaire contained questions about participants’ gender identity, sexual and/or affectional orientation, age, race, ethnicity, highest level of completed education, and primary role in the university community.

### 2.4. Data Analysis

To continue the work done by S. Zhang et al. (2025), we collected data from a new group of participants in a university community. We used both Classical Test Theory (CTT, Novick, 1966) and Item Response Theory (IRT, Hambleton et al., 1991) for the data analytic techniques. CTT is an effective guide to assess the reliability of scores and evidence of validity (López-Pina & Veas, 2024), and IRT supports the determination of item-level fit statistics (Aybek, 2023; C. Zhang et al., 2023).

The data analysis was completed in multiple steps. First, the opposite-statement items were removed from the data prior to analysis. Second, descriptive statistics were used to analyze the demographic data and provide an overview of participant characteristics. Third, we evaluated the skewness and kurtosis values to detect outliers for the CRI items (Dimitrov, 2012). The skewness values for all 47 items ranged from −0.657 to 0.228, and the kurtosis values ranged from −0.458 to 1.721. These results indicate that the data distribution for all items falls within the acceptable range of [−2, +2]. This suggests that the raw data did not violate the normal distribution assumption and there were no extreme cases/outliers. The distributions are well-suited for analyses that assume normality (Field, 2018; Tabachnick & Fidell, 2019; Kline, 2023; Hair et al., 2022).

Fourth, descriptive statistics were obtained for all the CRI items to provide overall perceptions of the participants at the item level. Fifth, a second-order CFA was conducted to assess the factor structure of the CRI. The six-factor structure was further analyzed to determine if all the first-order factors (i.e., *Efforts, Knowledge of Efforts, Leadership, Community Climate, Knowledge of Issue,* and *Resources*) collectively formed a second-order latent construct—*Community Readiness*. McDonald’s omega values and confidence intervals were determined as evidence of internal consistency. Finally, we used Rasch analysis to explore the item-level fit statistics for each subscale. Rasch analysis provided the mean square error (MSE) and standardized model-data fit statistics—infit and outfit (Bond & Fox, 2015; Boone et al., 2014; Engelhard, 2013; DeMars, 2023). For interpretation purposes, we utilized an MSE of 1.00 and standardized fit statistics within −2 and +2 as indications of good fit (Linacre, 2002).

## 3. Results

Our goals for this study were to confirm the (a) six-factor structure of CRI, (b) collective formation of the six first-order latent factors as the second-order latent construct—*Community Readiness*, (c) reliability of scores and validity evidence of the CRI, and (d) item-level fit statistics of the CRI items. All values suggested acceptable to great internal consistency.

### 3.1. Descriptive Statistics

We calculated the descriptive statistics of all 47 CRI items (Table 2). Most items had average ratings in the upper 2.00s and 3.00s, and the standard deviations were small across all items. These results indicate that participants demonstrated high and concentrated levels of agreement toward CRI items. For instance, the highest-rated CRI item was KI-9 (“the community members recognize that some students may be using substances,” *M* = 3.21, *SD* = 0.59), and the lowest-rated item was KE-6 (“the community members have specific knowledge about the effort(s),” *M* = 2.16, *SD* = 0.73).

### 3.2. Second-Order Confirmatory Factor Analysis Factor Loadings

**Figure 1:**
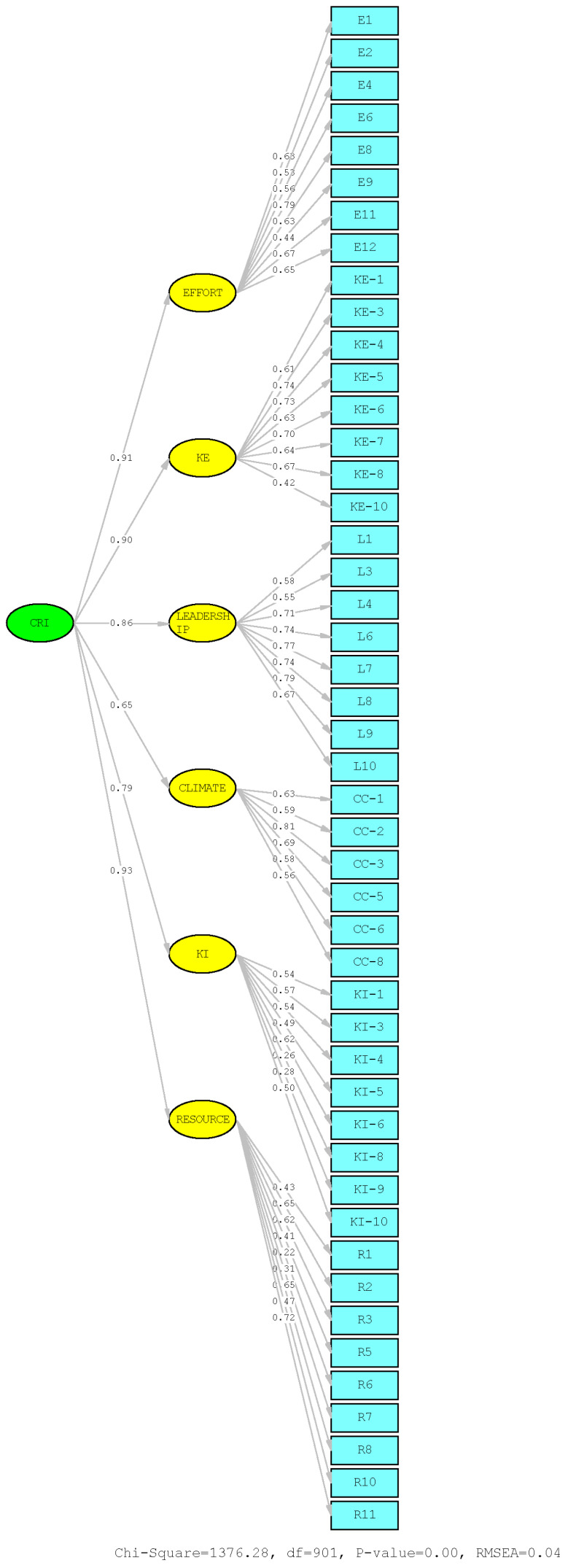
Path Diagram for CRI Second-Order CFA made using LISREL14. *Note.* Items KI-8, KI-9, R-6, and R-7 demonstrated weak factor loadings.

All items demonstrated satisfactory factor loadings, indicating that each item loaded strongly on the corresponding first-order latent factor (Figure 1). Standard instrumentation resources state that a factor loading above 0.3 is acceptable (Kline, 2015, 2023; Tabachnick & Fidell, 2019; Hair et al., 2022). Of the 47 CRI items, only three displayed factor loadings below that: KI-8 (0.26) and KI-9 (0.28) in the *Knowledge of Issue* subscale, and R-6 (0.22) in the *Resources* subscale. These items warranted further attention and discussion.

Both KI-8 and KI-9 assess participants’ knowledge of students in the university community using substances. This focus differs slightly from the other subscale items, which assess participants’ knowledge about the issue of substance use (e.g., harmful effects, signs and symptoms of use, benefits of treatment, etc.). The mean scores for KI-8 and KI-9 were higher than the other items, indicating stronger agreement. This may suggest participant confusion about those items compared to the other subscale items. It is also possible that this study’s participants (i.e., majority undergraduates at a four-year residential campus) have high collective awareness about the prevalence of student substance use because it is part of their lived experience. The other item concerns the availability of physical spaces (R-6) for the community to address student substance use. We determined that a sample consisting primarily of university undergraduates may not have access to information about campus facilities and personnel.

Awareness that an issue exists and knowledge of logistical resources are demonstrated components that inform community initiatives toward sustainable change (Tri-Ethnic Center for Prevention Research [TEC], 2025); therefore, assessing them is crucial. Although the observed loadings fell only slightly below the acceptable threshold (Hair et al., 2022; Kline, 2015, 2023), instrument development experts recommend that researchers interpret the 0.30 cutoff with flexibility, especially when items capture theoretically central aspects of the construct (Tabachnick & Fidell, 2019). Items KI-8, KI-9, and R-6 may reflect the community context and sample rather than psychometrically weak items (Boateng et al., 2018; Clark & Watson, 2019) that will fail to load significantly for most or all samples. For these reasons, we decided to retain them at this time to allow for further empirical testing.

A second-order CFA was conducted to determine if the six first-order CRI factors collectively form a second-order latent construct of *Community Readiness*. The initial model modification indices suggested that a number of error covariances were correlated. After improving the model, the revised second-order CFA results indicated that the six-factor solution was an excellent fit for the hypothesized measurement model (Figure 1). All the CFA model fit statistics indicated that CRI is a tool with great evidence of validity: (*RMSEA*) = 0.037, (*SRMR*) = 0.056, (*CFI*) = 0.990, (*TLI*) = 0.967, and the normed fit index (*NFI*) = 0.960.

Similar to S. Zhang et al. (2025), we also conducted a reliability analysis using McDonald’s omega values (McDonald, 1999). The items within each subscale fell into one-factor solutions, with omega values in the “great” to “excellent” range (Dunn et al., 2014; Hayes & Coutts, 2020; McDonald, 1999): *Efforts* ω = 0.86 (95% CI = 0.84, 0.89), *Knowledge of Efforts* ω = 0.88 (95% CI = 0.87, 0.91), *Leadership* ω = 0.92 (95% CI = 0.91, 0.94), *Community Climate* ω = 0.89 (95% CI = 0.87, 0.92), *Knowledge of Issue* ω = 0.82 (95% CI = 0.80, 0.86), and *Resources* ω = 0.85 (95% CI = 0.81, 0.88). These results indicate that the CRI is a psychometrically strong measure of each community readiness dimension represented by the CRI subscales.

### 3.3. Rasch Analysis

We used *Facets* (Linacre, 2024) to perform the Rasch analysis. Rasch fit statistics determined that most CRI items fell within the productive range of 0.5–1.5 (Bond et al., 2020; Boone, 2016; DeMars, 2023; Linacre, 2002). The only item that warranted attention was L-1 in the *Leadership* subscale, which had an Infit statistic of 1.51, slightly above the upper bound. We agreed to retain the item for future testing, as the fit statistic did not deviate from the productive threshold. We also believe the information gathered by this item—“Community leaders view student substance use as a major concern”—contributes crucial information in this subscale.

We also chose to examine the Differential Item Functioning (DIF) for all six CRI subscales using many-facet Rasch models. DIF evaluates the measurement invariance to determine if the items function equivalently across subgroups (Bond & Fox, 2015; DeVellis, 2016; Effatpanah et al., 2025). We calculated the DIF for participants’ reported age group and their primary role in the university community (students or faculty/staff/administration).

The results across the six subscales were consistent for the age group; model-level DIF tests were not significant (fixed effects χ^2^ tests, *p* > 0.05), and age facet separation indices were near zero, indicating negligible age-related differences in item functioning. Similarly, item-by-primary role interaction estimates were small in magnitude, with DIF contrasts well below ±0.30 logits. Associated standardized statistics failed to reach significance thresholds. Finally, item fit statistics remained within accepted Rasch ranges across these groups. Although these results provide statistical support for measurement invariance for the CRI across age groups and primary roles, assessing group differences was not the focus of this full-scale instrumentation study. The full DIF analysis of measurement invariance and meaningful differences between subgroups will be provided in a forthcoming manuscript (Ricciutti et al., 2026).

### 3.4. The Community Readiness Scores

Although not one of our research questions, we also calculated each subscale’s means and standard deviations of the overall scores. Participants’ ratings across all six CRI subscales were fairly consistent, with small standard deviations. The *Knowledge of Issue* subscale had the highest mean score (*M* = 2.84, *SD* = 0.38), followed by *Community Climate* (*M* = 2.76, *SD* = 0.47), *Leadership* (*M* = 2.68, *SD* = 0.50), *Efforts* (*M* = 2.63, *SD* = 0.46), *Resources* (*M* = 2.55, *SD* = 0.40), and *Knowledge of Efforts* (*M* = 2.43, *SD* = 0.51). The university community may have been in *Contemplation* or *Preparation* in each dimension, as a score of 2 on the Likert scale was indicative of *Contemplation* and a score of 3 was indicative of *Preparation*.

## 4. Discussion

Researchers and community advocates have repeatedly called for a way to easily measure community change readiness (Cureton et al., 2022; Hicks, 2019; Kelley et al., 2018; Kostadinov et al., 2015). Although the CRI showed promising initial psychometrics (S. Zhang et al., 2025), additional tests were necessary to confirm the original results. The goals of the present study were to further test the psychometric properties of the CRI and to determine its validity using second-order CFA and Rasch analyses. Our overarching intention was to ensure the CRI’s effectiveness for measuring the overall concept of *Community Readiness* and its associated readiness dimensions/subscales: *Efforts, Knowledge of Efforts, Leadership, Community Climate, Knowledge of Issue,* and *Resources*.

The findings show that the CRI functions well as a statistical measure for assessing community readiness. Results from the present study confirmed the overall factor structure of the CRI that was found in the pilot study (S. Zhang et al., 2025). Specifically, the six factors/subscales collectively form a higher-order construct: Community Readiness. At the item level, we tested the CRI items to ensure that they strongly resembled their corresponding factors. The overall factor structure did not change from the initial testing; however, four items demonstrated weak factor loadings. We recommend additional testing to further determine whether or not to keep or alter those items. We discuss implications for using the CRI as a quantitative assessment of community readiness and directions for ongoing evaluation and advancement of the measure.

### 4.1. Implications for Community-Engaged Researchers

This follow-up test of the CRI highlighted numerous implications for community readiness assessment, namely, maximizing its adaptability and statistical soundness to benefit various communities and issues. Although the pilot and current study targeted the issue of college student substance use in a university community, both appear usable for a variety of issues and communities (S. Zhang et al., 2025) based on our choice to alter item-level phrasing to more inclusive terms (i.e., “The community…” instead of “XU community…”).

The CRI developers recommended ongoing exploration of construct validity through applications to diverse communities and issues (S. Zhang et al., 2025). For example, the CRM (Plested et al., 2016; Tri-Ethnic Center for Prevention Research [TEC], 2014) has been used in Black/African American communities (Cureton et al., 2023; James et al., 2022) and Native/Indigenous communities (Hunter, 2024), university communities (Chilenski et al., 2007; Swan & Allan, 2023), and specific professional communities (Castañeda et al., 2014; Carter-Veale et al., 2025; Cureton et al., 2020). The CRI can be used to identify and improve low readiness within a single community or across several communities. The CRM (Plested et al., 2016; Tri-Ethnic Center for Prevention Research [TEC], 2014) has been applied to various issues such as suicide (Cureton et al., 2020, 2023), childhood obesity (Heath et al., 2020; Schröder et al., 2022), intimate partner violence (K. M. Edwards et al., 2016; Yoshihama & Hammock, 2023), and substance use (Ghahremani et al., 2021). The CRI can now be leveraged for meaningful research and prevention to gather quantitative data on these and other issues from large community samples.

The CRI developers (S. Zhang et al., 2025) worked to retain many of the TTM’s (Prochaska & DiClemente, 1982) and CRM’s strengths (Tri-Ethnic Center for Prevention Research [TEC], 2025). The CRM manuals recommend that users define the target community and issue at the start of each interview (Plested et al., 2016; Tri-Ethnic Center for Prevention Research [TEC], 2014). Accordingly, the CRI (S. Zhang et al., 2025) includes definitions of the community and issue in the instructions and at the start of each subscale. S. Zhang et al. (2025) encouraged CRI users to follow the recommended action steps for all dimensions/subscales that appear in the CRM Manuals (Tri-Ethnic Center for Prevention Research [TEC], 2025) as the CRI subscales and score ranges were developed to reflect the CRM’s dimensions of community readiness (Plested et al., 2016; Tri-Ethnic Center for Prevention Research [TEC], 2014, 2025) and the TTM’s stages of change (Prochaska & DiClemente, 1982).

As an example, CRI scores in the *Action* stage for the *Knowledge of Efforts* subscale and the *Contemplation* stage for the *Leadership* subscale may indicate that community members are aware of existing efforts to address the target issue, while leaders may lack interest in enacting change. Advocates and researchers can take steps to increase *Leadership* readiness by (a) presenting information at city council meetings, (b) hosting events related to the issue and inviting community leaders, and (c) holding meetings with leaders to invest them in the changes being made. Maintaining high scores in *Knowledge of Efforts* can involve (a) training healthcare professionals about the issue, (b) planning publicity events for the existing or upcoming efforts, and (c) conducting quarterly meetings to review progress and modify existing strategies. The CRI’s demonstrated properties also support its prospective use for pre-/post-comparisons to determine an intervention’s effectiveness for increasing the community’s dimensional and/or overall readiness. Using the CRI alongside the CRM to gather well-rounded and rigorous mixed-method community data can also inform ongoing action planning (S. Zhang et al., 2025).

### 4.2. Limitations and Directions for Future CRI Research

This study relied on self-reported perceptions of community readiness. Participants’ responses may have been subject to social desirability response bias and personal attitudes about the issue of student substance use. We determined that the 19 opposite-statement items were unnecessary, as neither our study nor S. Zhang et al.’s (2025) study found cause to remove participants. We recommend that future researchers exclude the opposite-statement items and use only the 47 items analyzed.

The study’s purpose was to determine the CRI’s psychometric properties. The CFA highlighted three items warranting further attention: two in the *Knowledge of Issue* subscale and one in the *Resources* subscale. We evaluated any perceptual differences between subgroups (by age and primary role only), then retained these items because subgroup evaluations showed no meaningful differences, and the items measure important aspects of community readiness. The majority of participants identified as students from one university in the southeastern U.S. It is an accepted practice to initiate psychometric testing and tool validation with a college student sample and/or in a university setting (Bond & Fox, 2015; DeVellis, 2016; DeVellis & Thorpe, 2021; Marakshina et al., 2025). The current setting and sample seem a logical fit for this initial assessment on college student substance use, and college students likely have intimate awareness of their own and other students’ use compared to other members of the university community. Nevertheless, the current sample could limit generalizability to other settings, and weak loadings might be attributable to the initial community setting and/or sample (Boateng et al., 2018; Clark & Watson, 2019; Wild et al., 2022). For these reasons, we encourage future researchers to test the CRI within and across a variety of populations and diverse community contexts to assess its evidence of construct validity and external validity. Should items consistently demonstrate weak loadings or poor item functioning, we recommend removal or substantial revision. This may include the meaningful development of a short form for abbreviated assessment and/or retest purposes.

Community-engaged research and CRM studies frequently take place in settings where community members may not know all of the details of the community’s change efforts (K. M. Edwards et al., 2016; Swan & Allan, 2023). This lack of specific knowledge can actually inform policy efforts and point toward areas of growth for local advocates (Schröder et al., 2022; Tri-Ethnic Center for Prevention Research [TEC], 2014, 2025; Wainwright et al., 2024). Another promising direction involves using the CRI with other assessments. We highly encourage future researchers to conduct studies with the CRI alongside the CRM (Tri-Ethnic Center for Prevention Research [TEC], 2025) in mixed-methods research. The CRI is based on the CRM, and both have a strong theoretical base in the TTM (Prochaska & DiClemente, 1982). Using both may provide rich insights into a community’s change readiness toward a specific issue. CRM findings might specify or explain CRI results, and vice versa. For example, if a community’s CRI results include low scores in the *Efforts* and *Resources* subscales, the CRM findings can provide helpful context. Budding research has involved rigorous qualitative research principles applied to CRM interview data (Cureton et al., 2022, 2023; James et al., 2022). This process can serve as evidence of the CRI’s effectiveness as a standalone tool or whether it should be used in conjunction with the CRM. Similarly, studies evaluating the utility of the CRI with TTM assessments of individual readiness are another promising direction for future research and may be feasible given the overlapping score range structures between the two.

We estimated the community’s change readiness may be between *Contemplation* and *Preparation* based on the means and standard deviations of each CRI subscale. Scale development literature supports the use of these figures for such conclusions (Olsen et al., 2020; Stone & Dahir, 2015); however, more definitive CRI scoring procedures are still needed. Researchers can develop scoring instructions to guide future CRI use for diverse community prevention initiatives. Finally, a key application of the CRI is its potential to guide policy changes. Future researchers should explore whether using CRI data to inform community action plans can lead to significant and measurable improvements in readiness and systemic behavioral change. Researchers may also assess whether or not the increased perception of CRI positively correlated with successful policy adoption and ongoing community impact.

## 5. Conclusions

We addressed three research questions in this study to further determine the CRI’s psychometric properties and evidence of its validity. The first question concerned the reliability of the CRI subscales. Each subscale displayed “great” to “excellent” McDonald’s Omega values. For the second research question, results from the second-order CFA and Rasch analyses confirmed that CRI showed sound structural integrity and could reliably measure the six dimensions of community readiness. For the third research question on CRI psychometrics via Rasch analysis, fit statistics demonstrated that the CRI items were productive measures of each subscale. Our findings aligned with previous research (S. Zhang et al., 2025) and introduced a long-awaited quantitative community readiness assessment tool. The findings of this study contributed substantially to the current community readiness field by promoting the CRI’s evidence of validity and its use as a psychometrically robust quantitative measure of community readiness.

## Figures and Tables

**Table 1:** Community Readiness Tools.

Tools	Attributes							
	EFA	CFA	Rasch	Expert Review	TTM	CRM	SUD	Issue Adapt.
CRA (Plested et al., 2016)				X	X	X	X	X
CCRS (Hicks, 2019)				X		X		X
RAP-CM (Mikton et al., 2013)				X				
RAP-CM Short Version (WHO, 2013)				X				
SOC-RIMS (Rosas et al., 2016)	X			X				
CRS (Beebe et al., 2001)	X			X	X	X		
CRI (S. Zhang et al., 2025)	X	X	X	X	X	X	X	X

*Notes:* EFA = Reliability of subscale scores determined through exploratory factor analysis. CFA = Psychometric properties and evidence of validity determined through confirmatory factor analysis. Rasch = Item-level fit statistics determined through Rasch analysis. TTM = The Transtheoretical Model of Change is the theoretical foundation for the measure. CRM = The measure was developed based on the CRM. SUD = Previous application(s) to SUD or related target issue. Issue adapt. = Usable or adaptable to more than one target issue.

**Table 2:** Descriptive Statistics and Rasch Analysis Results of CRI items.

Items	*M*	*SD*	*Measure*	*Infit*	*Outfit*
E-1. The community members view the current effort(s) to address substance use as widely successful.	2.44	0.67	0.66	0.91	0.89
E-2. The effort(s) in the community to address student substance use have existed for a long time (i.e., 4 or more years).	2.82	0.68	−0.72	1.03	1.03
E-4. The effort(s) in the community to address student substance use serve a broad range of students (i.e., racial/ethnic, sexual orientation, first-generation students, veteran students, etc., groups).	2.84	0.75	−0.80	1.27	1.26
E-6. There are many efforts in the community to address student substance use	2.45	0.75	0.60	0.86	0.84
E-8. There are many in the community (e.g., individuals, university offices and departments) who are trying to get something started to address student substance use.	2.58	0.68	0.17	0.91	0.90
E-9. Most community members see a need for efforts to address student substance use.	2.77	0.67	−0.52	1.23	1.23
E-11. Meetings have been held in the community (e.g., student groups, departments, or university offices) to discuss the effort(s) to address student substance use.	2.62	0.68	0.08	0.85	0.83
E-12. Evaluations valuation plans are used to test the effectiveness of the community effort(s) to address student substance use.	2.50	0.65	0.53	0.91	0.90
KE-1. The community members have accurate information about the effort(s) to address student substance use.	2.44	0.70	−0.07	1.15	1.11
KE-3. The community members have heard of the effort(s) to address student substance use.	2.58	0.73	−0.53	0.91	0.90
KE-4. The community members can name the effort(s) to address student substance use.	2.17	0.77	1.05	0.91	0.92
KE-5. The community members have basic knowledge about the effort(s) to address student substance use. (Basic knowledge might include knowing the purpose of the efforts or who the efforts are for.)	2.70	0.67	−1.17	0.98	0.93
KE-6. The community members have specific knowledge about the effort(s). (Specific knowledge might include when and where the efforts occur, how they are implemented, etc.).	2.16	0.73	1.12	0.79	0.80
KE-7. The community members know how well (or not well) the effort(s) to address student substance use are working.	2.19	0.72	0.94	0.90	0.90
KE-8. The community members are aware of the strengths/benefits of the effort(s) to address student substance use.	2.64	0.72	−0.84	1.13	1.10
KE-10. The community members are aware of the weaknesses/limitations of the effort(s) to address student substance use.	2.58	0.66	−0.49	1.22	1.23
L-1. The community leaders view student substance use as a major concern.	2.72	0.77	−0.26	1.51	1.43
L-3. The community leaders believe student substance use is a problem	2.91	0.64	−1.25	1.16	1.18
L-4. The community leaders view addressing the issue of student substance use as a major priority.	2.44	0.73	1.20	0.98	0.95
L-6. The community leaders actively support the effort(s) to address student substance use.	2.78	0.61	−0.55	0.82	0.79
L-7. The community leaders have participated in developing, improving, or implementing the effort(s) to address student substance use.	2.66	0.64	0.12	0.76	0.72
L-8. The community leaders are seeking or allocating resources to fund efforts to address student substance use.	2.53	0.67	0.81	0.87	0.86
L-9. The community leaders are driving initiatives to improve/expand the effort(s) to address student substance use.	2.54	0.66	0.74	0.79	0.74
L-10. The community leaders support the development of new efforts to address the issue of student substance use.	2.83	0.58	−0.81	0.94	0.90
CC-1. The community members believe that student substance use is a concern.	2.87	0.61	−0.61	0.82	0.80
CC-2. The community members believe the issue of student substance use should be addressed.	2.97	0.57	−1.17	0.88	0.89
CC-3. Addressing the issue of student substance use is a priority to the community members.	2.58	0.70	0.97	0.88	0.86
CC-5. The community members actively support the effort(s) to address student substance use.	2.75	0.62	0.10	1.06	1.09
CC-6. The community members support expanding the existing effort(s) to address student substance use.	2.81	0.57	−0.25	0.96	1.07
CC-8. The community members seem troubled by student substance use.	2.58	0.70	0.97	1.19	1.26
KI-1. Members of the community have access to information regarding student substance use.	2.66	0.75	0.71	1.24	1.25
KI-3. Members of the community have basic knowledge about student substance use (e.g., signs and symptoms that someone is using).	2.91	0.61	−0.24	0.77	0.73
KI-4. The community members have specific knowledge about student substance use (why students use, how many/often students use, impacts of and treatments for using, etc.).	2.32	0.71	1.88	1.14	1.21
KI-5. The community members know the harmful effects of student substance use.	3.07	0.57	−0.87	0.80	0.80
KI-6. The community members have accurate information about student substance use.	2.41	0.67	1.61	0.86	0.89
KI-8. The community members know that student substance use occurs on campus.	3.18	0.65	−1.31	1.20	1.26
KI-9. The community members recognize that some students may be using substances.	3.21	0.59	−1.48	1.00	1.07
KI-10. The community members consider student substance use to be worth knowing about.	2.93	0.59	−0.29	0.94	0.93
R-1. There are many resources in the community that could be used to address student substance use (even if they are not currently used for that issue).	2.69	0.72	−0.48	1.26	1.23
R-2. The existing effort(s) to address student substance use in the community are adequately funded.	2.34	0.64	0.77	0.75	0.75
R-3. Substantial financial resources (e.g., budgets, donations, grants, etc.) are available to members of the community to address student substance use.	2.21	0.66	1.24	0.78	0.78
R-5. There is plenty of time available to the community (e.g., to plan and implement efforts) to address student substance use.	2.63	0.70	−0.24	1.12	1.08
R-6. There are plenty of places/locations available in the community to host efforts to address student substance use.	2.92	0.64	−1.34	1.11	1.06
R-7. There are plenty of people available to the community (e.g., to coordinate or volunteer for efforts) to address student substance use.	2.81	0.65	−0.88	1.04	1.00
R-8. Many experts on student substance use are invited to campus to inform the community about the issue.	2.19	0.69	1.35	1.03	1.03
R-10. Members of the community support using available resources for the effort(s) to address student substance use.	2.86	0.57	−1.11	0.95	0.93
R-11. There are numerous initiatives by the community to seek additional resources (e.g., money, time, space, people) for the effort(s) to address student substance use.	2.37	0.69	0.69	0.90	0.91

*Note.* Items 3, 5, 7, 10, and 13 for *Efforts*, items 2, 9, and 11 for *Knowledge of Issue*, items 2 and 5 for *Leadership*, items 4, 7, and 9 for *Community Climate*, items 2, 7, and 11 for *Knowledge of Efforts*, and items 4, 9, and 12 for *Resources* were opposite-statement items, and thus were excluded from data analyses. Infit is defined as an information-weighted mean square statistic; Outfit is defined as an outlier-sensitive mean square statistic.

## Data Availability

The data for this study is not publicly available.
